# Bis[aqua­(2,3-naphtho-15-crown-5)sodium] tetra­kis(thio­cyanato-κ*N*)cobaltate(II)

**DOI:** 10.1107/S1600536809038641

**Published:** 2009-10-07

**Authors:** Chengjuan Li, Da-Cheng Li, Da-Qi Wang

**Affiliations:** a College of Chemistry and Chemical Engineering, Liaocheng University, Shandong 252059, People’s Republic of China

## Abstract

The title complex, [Na(C_18_H_22_O_5_)(H_2_O)]_2_[Co(NCS)_4_], consists of two aqua­(2,3-naphtho-15-crown-5)sodium complex cations and one [Co(NCS)_4_]^2−^ complex anion, which has crystallographic 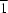 symmetry. In the anion, the Co^II^ centre is coordinated by the N atoms of four NCS^−^ ligands in a distorted tetra­hedral geometry. In the complex cations, the Na^I^ centre is coordinated by five O atoms of the 2,3-naphtho-15-crown-5 ligand and one water O atom. The complex mol­ecules form a two-dimensional network *via* weak O—H⋯S inter­actions between adjacent cations and anions

## Related literature

For crown ether complexes, see: Pedersen (1967 ▶); Zhang *et al.* (1996 ▶). For π–π inter­actions of the naphtho crown ether, see: Gao *et al.* (2005 ▶). For structural information on compounds with similar features, see Fan *et al.* (1985 ▶); Dou *et al.* (2004 ▶); Yu *et al.* (2005 ▶); Zhang *et al.* (2006 ▶).
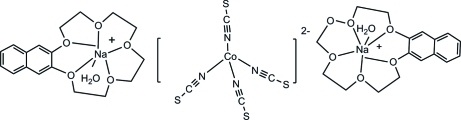


## Experimental

### 

#### Crystal data

[Na(C_18_H_22_O_5_)(H_2_O)]_2_[Co(NCS)_4_]
*M* *_r_* = 1009.97
Monoclinic, 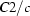

*a* = 23.030 (5) Å
*b* = 13.170 (3) Å
*c* = 17.469 (4) Å
β = 115.255 (3)°
*V* = 4792.0 (18) Å^3^
*Z* = 4
Mo *K*α radiation
μ = 0.61 mm^−1^
*T* = 298 K
0.39 × 0.36 × 0.18 mm

#### Data collection

Bruker SMART CCD area-detector diffractometer
Absorption correction: multi-scan (*SADABS*; Sheldrick, 1996 ▶) *T*_min_ = 0.796, *T*_max_ = 0.898
12300 measured reflections
4232 independent reflections
2150 reflections with *I* > 2σ(*I*)
*R*_int_ = 0.050

#### Refinement

*R*[*F* ^2^ > 2σ(*F* ^2^)] = 0.046
*wR*(*F* ^2^) = 0.110
*S* = 1.00
4232 reflections
285 parameters
6 restraints
H-atom parameters constrained
Δρ_max_ = 0.25 e Å^−3^
Δρ_min_ = −0.28 e Å^−3^

### 

Data collection: *SMART* (Siemens, 1996 ▶); cell refinement: *SAINT* (Siemens, 1996 ▶); data reduction: *SAINT*; program(s) used to solve structure: *SHELXS97* (Sheldrick, 2008 ▶); program(s) used to refine structure: *SHELXL97* (Sheldrick, 2008 ▶); molecular graphics: *SHELXTL* (Sheldrick, 2008 ▶); software used to prepare material for publication: *SHELXTL*.

## Supplementary Material

Crystal structure: contains datablocks I, global. DOI: 10.1107/S1600536809038641/pk2189sup1.cif

Structure factors: contains datablocks I. DOI: 10.1107/S1600536809038641/pk2189Isup2.hkl

Additional supplementary materials:  crystallographic information; 3D view; checkCIF report

## Figures and Tables

**Table 1:** Hydrogen-bond geometry (Å, °)

*D*—H⋯*A*	*D*—H	H⋯*A*	*D*⋯*A*	*D*—H⋯*A*
O6—H1⋯S2^i^	0.84	2.62	3.458 (3)	173
O6—H3⋯S1^ii^	0.84	2.57	3.389 (3)	165

Symmetry codes: (i) 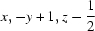; (ii) 
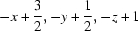
.

**Fig. 1.**
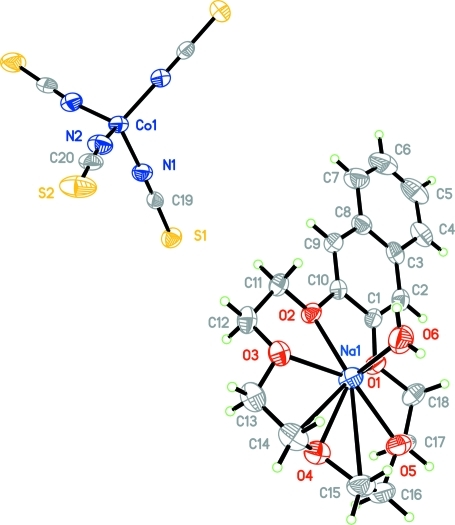
The molecular structure of the compound, showing 40% probability displacement ellipsoids.

**Fig. 2.**
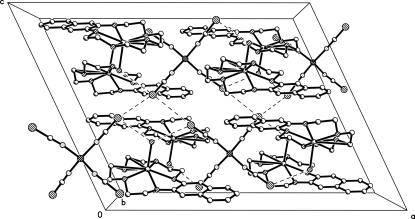
View of the two-dimensional network structure in the crystal structure of the title complex. Intermolecular O—H···S are shown. H atoms have been omitted.

## supplementary crystallographic information

### Comment

Due to the unusual coordination numbers and arrangements with various metal ions, crown ethers have attracted much attention (Pedersen, 1967; Zhang *et al.*, 1996). In particular, the naphtho crown ethers play an important role in crystal engineering of supramolecular interactions, such as π···π interactions (Dou *et al.*, 2004; Gao *et al.*, 2005). In order to study the weak interactions of the naphtho crown ether, we have synthesized the title complex by the reaction of naphtho-15-crown-5 with CoCl_2_ and NaSCN. The title complex, [Na(N15C5)H_2_O]_2_[Co(NCS)_4_] is made up of two [Na(N15C5)H_2_O]^+^ complex cations and one [Co(NCS)_4_]^2-^ complex anion. In the complex anion, the Co is coordinated by four N atoms of the NCS ligands and exhibits a distorted tetrahedral geometry. The average Co—N bond length is 1.945 (4) Å, which is consistent with that of [K(18C6)]_2_[Co(SCN)_4_](1.94 Å) (Fan *et al.*, 1985). In the complex cation, the Na is coordinated by five O atoms of the crown ether and one O atom of the H_2_O, The Na—O(crown ether) distances are in the range 2.329 (3) to 2.361 (3) Å), average 2.344 (3) Å, which is shorter than that of [Na(N15C5)][Pd(SCN)_4_] (Dou *et al.*, 2004). The Na—O(H_2_O) bond length is 2.278 (3) Å, which is comparable to that of [{Na(18C6)}_2_(H_2_O)][Ni(i-mnt)_2_] (Yu *et al.*, 2005). For the neighboring complex cations and the complex anions, the distances of O(6)···S(2) and O(6)—S(1) are 3.46 (3) Å and 3.39 (4) Å respectively, which are shorter than those in the complex {[(Me_3_Sn)(µ-OH)][Me_2_Sn(µ_3_-SCH_2_CO_2_)]}_n_ (Zhang *et al.*, 2006), indicating that there are O—H···S weak interactions between the neighboring complex cations and complex anions. The title complex is assembled into two-dimensional network by virtue of O—H···S weak interactions.

### Experimental

The title complex was prepared by adding 10 ml of an aqueous mixture of CoCl_2_ (0.032 g, 0.25 mmol) and NaSCN (0.81 g, 10 mmol) to a solution of 2,3-naphtho-15-crown-5 (0.16 g, 0.5 mmol) in 10 ml dichloromethane. The reaction mixture was stirred for 2 hrs at room temperature and filtered. The precipite was dissolved in the CH_3_CN, and the crystal was obtained a week later. m.p.: 470–472. Anal. Calcd (%) for C_40_H_48_CoN_4_Na_2_O_12_S_4_: C, 47.45; H, 4.82; N, 5.62. Found (%): C, 47.52; H, 4.75; N, 5.54.

### Refinement

The C—H H atoms were positioned with idealized geometry and were refined as isotropic with *U*_iso_(H) = 1.2 *U*_eq_(C, O) for all H atoms using a riding model with C—H = 0.93 Å for aromatic H, and C—H = 0.97 Å for CH_2_. The S2 atom was refined using an approximately isotropic model (ISOR in SHELXL).

### Crystal data

**Table:**

[Na(C_18_H_22_O_5_)(H_2_O)]_2_[Co(NCS)_4_]	*F*(000) = 2100
*M**_r_* = 1009.97	*D*_x_ = 1.400 Mg m^−^^3^
Monoclinic, *C*2/*c*	Mo *K*α radiation, λ = 0.71073 Å
Hall symbol: -C 2yc	Cell parameters from 1972 reflections
*a* = 23.030 (5) Å	θ = 2.5–21.9°
*b* = 13.170 (3) Å	µ = 0.61 mm^−^^1^
*c* = 17.469 (4) Å	*T* = 298 K
β = 115.255 (3)°	Block, red
*V* = 4792.0 (18) Å^3^	0.39 × 0.36 × 0.18 mm
*Z* = 4	

### Data collection

**Table:**

Bruker SMART CCD area-detector diffractometer	4232 independent reflections
Radiation source: fine-focus sealed tube	2150 reflections with *I* > 2σ(*I*)
graphite	*R*_int_ = 0.050
φ and φ scans	θ_max_ = 25.0°, θ_min_ = 1.8°
Absorption correction: multi-scan (*SADABS*; Sheldrick, 1996)	*h* = −23→27
*T*_min_ = 0.796, *T*_max_ = 0.898	*k* = −15→10
12300 measured reflections	*l* = −20→20

### Refinement

**Table:**

Refinement on *F*^2^	Primary atom site location: structure-invariant direct methods
Least-squares matrix: full	Secondary atom site location: difference Fourier map
*R*[*F*^2^ > 2σ(*F*^2^)] = 0.046	Hydrogen site location: inferred from neighbouring sites
*wR*(*F*^2^) = 0.110	H-atom parameters constrained
*S* = 1.00	*w* = 1/[σ^2^(*F*_o_^2^) + (0.0212*P*)^2^ + 7.4253*P*] where *P* = (*F*_o_^2^ + 2*F*_c_^2^)/3
4232 reflections	(Δ/σ)_max_ = 0.001
285 parameters	Δρ_max_ = 0.25 e Å^−^^3^
6 restraints	Δρ_min_ = −0.28 e Å^−^^3^

### Special details

**Table:**

Refinement. Refinement of *F*^2^ against ALL reflections. The weighted *R*-factor *wR* and goodness of fit *S* are based on *F*^2^, conventional *R*-factors *R* are based on *F*, with *F* set to zero for negative *F*^2^. The threshold expression of *F*^2^ > σ(*F*^2^) is used only for calculating *R*-factors(gt) *etc*. and is not relevant to the choice of reflections for refinement. *R*-factors based on *F*^2^ are statistically about twice as large as those based on *F*, and *R*- factors based on ALL data will be even larger.

### Fractional atomic coordinates and isotropic or equivalent isotropic displacement parameters (Å^2^)

**Table:**

	*x*	*y*	*z*	*U*_iso_*/*U*_eq_	
Co1	0.5000	0.51700 (7)	0.7500	0.0623 (3)	
Na1	0.81291 (7)	0.28279 (13)	0.32041 (10)	0.0657 (5)	
N1	0.57146 (16)	0.4339 (3)	0.8270 (2)	0.0680 (10)	
N2	0.53079 (17)	0.5994 (3)	0.6834 (3)	0.0808 (12)	
O1	0.80689 (12)	0.1597 (2)	0.41499 (16)	0.0580 (7)	
O2	0.73752 (11)	0.3173 (2)	0.37301 (15)	0.0534 (7)	
O3	0.77894 (13)	0.4521 (2)	0.28951 (16)	0.0632 (8)	
O4	0.90108 (13)	0.3783 (2)	0.32829 (17)	0.0680 (8)	
O5	0.90001 (12)	0.1712 (2)	0.36137 (18)	0.0690 (8)	
O6	0.75231 (14)	0.2494 (2)	0.18076 (19)	0.0952 (11)	
H1	0.7131	0.2636	0.1564	0.114*	
H3	0.7649	0.2381	0.1427	0.114*	
S1	0.67314 (6)	0.32131 (10)	0.94135 (7)	0.0739 (4)	
S2	0.58936 (6)	0.70155 (14)	0.59663 (10)	0.1164 (6)	
C1	0.74760 (18)	0.1464 (3)	0.4142 (2)	0.0472 (10)	
C2	0.72491 (19)	0.0597 (3)	0.4328 (2)	0.0558 (11)	
H2	0.7506	0.0020	0.4480	0.067*	
C3	0.6622 (2)	0.0558 (3)	0.4292 (2)	0.0580 (11)	
C4	0.6371 (2)	−0.0332 (4)	0.4494 (3)	0.0766 (14)	
H4	0.6617	−0.0920	0.4647	0.092*	
C5	0.5771 (3)	−0.0328 (5)	0.4463 (3)	0.0969 (18)	
H5	0.5613	−0.0916	0.4601	0.116*	
C6	0.5395 (3)	0.0523 (6)	0.4234 (3)	0.1004 (19)	
H6	0.4988	0.0508	0.4221	0.121*	
C7	0.5614 (2)	0.1392 (4)	0.4024 (3)	0.0862 (15)	
H7	0.5352	0.1963	0.3861	0.103*	
C8	0.62357 (19)	0.1435 (4)	0.4052 (3)	0.0596 (11)	
C9	0.64775 (18)	0.2321 (3)	0.3848 (2)	0.0579 (11)	
H9	0.6220	0.2898	0.3678	0.070*	
C10	0.70851 (18)	0.2349 (3)	0.3896 (2)	0.0459 (9)	
C11	0.69937 (19)	0.4050 (3)	0.3348 (3)	0.0606 (11)	
H11A	0.6762	0.4263	0.3672	0.073*	
H11B	0.6684	0.3891	0.2777	0.073*	
C12	0.7428 (2)	0.4878 (3)	0.3330 (3)	0.0668 (12)	
H12A	0.7177	0.5467	0.3042	0.080*	
H12B	0.7715	0.5073	0.3903	0.080*	
C13	0.8307 (2)	0.5175 (4)	0.2980 (3)	0.0770 (14)	
H13A	0.8541	0.5386	0.3564	0.092*	
H13B	0.8145	0.5777	0.2632	0.092*	
C14	0.8738 (2)	0.4593 (4)	0.2697 (3)	0.0778 (14)	
H14A	0.8495	0.4326	0.2132	0.093*	
H14B	0.9074	0.5031	0.2688	0.093*	
C15	0.9376 (2)	0.3071 (4)	0.3053 (3)	0.0813 (15)	
H15A	0.9754	0.3398	0.3055	0.098*	
H15B	0.9121	0.2808	0.2490	0.098*	
C16	0.9568 (2)	0.2230 (4)	0.3686 (3)	0.0842 (15)	
H16A	0.9851	0.1761	0.3580	0.101*	
H16B	0.9796	0.2502	0.4253	0.101*	
C17	0.91031 (19)	0.1114 (4)	0.4336 (3)	0.0762 (14)	
H17A	0.9302	0.1522	0.4845	0.091*	
H17B	0.9387	0.0551	0.4378	0.091*	
C18	0.84685 (19)	0.0722 (3)	0.4251 (3)	0.0689 (13)	
H18A	0.8279	0.0279	0.3762	0.083*	
H18B	0.8520	0.0343	0.4752	0.083*	
C19	0.61400 (19)	0.3861 (3)	0.8744 (3)	0.0535 (11)	
C20	0.55531 (19)	0.6421 (4)	0.6479 (3)	0.0719 (14)	

### Atomic displacement parameters (Å^2^)

**Table:**

	*U*^11^	*U*^22^	*U*^33^	*U*^12^	*U*^13^	*U*^23^
Co1	0.0459 (5)	0.0642 (6)	0.0740 (6)	0.000	0.0228 (4)	0.000
Na1	0.0605 (10)	0.0661 (12)	0.0759 (11)	0.0079 (8)	0.0344 (9)	0.0094 (9)
N1	0.052 (2)	0.066 (3)	0.084 (3)	−0.0012 (19)	0.027 (2)	0.006 (2)
N2	0.059 (2)	0.090 (3)	0.091 (3)	−0.008 (2)	0.030 (2)	0.013 (2)
O1	0.0525 (17)	0.0562 (19)	0.0705 (19)	0.0140 (14)	0.0311 (14)	0.0077 (14)
O2	0.0524 (16)	0.0422 (17)	0.0665 (18)	0.0095 (13)	0.0263 (14)	0.0088 (14)
O3	0.0724 (19)	0.056 (2)	0.0596 (18)	−0.0050 (15)	0.0268 (16)	−0.0006 (14)
O4	0.0646 (18)	0.088 (2)	0.0551 (18)	−0.0045 (17)	0.0291 (15)	−0.0010 (17)
O5	0.0531 (18)	0.083 (2)	0.072 (2)	0.0075 (16)	0.0281 (15)	−0.0001 (17)
O6	0.088 (2)	0.099 (3)	0.084 (2)	0.0186 (19)	0.0222 (19)	−0.0222 (19)
S1	0.0733 (8)	0.0708 (9)	0.0714 (8)	0.0126 (6)	0.0250 (6)	0.0094 (6)
S2	0.0666 (9)	0.1589 (17)	0.1127 (12)	−0.0027 (9)	0.0277 (8)	0.0643 (11)
C1	0.051 (2)	0.044 (3)	0.045 (2)	0.006 (2)	0.0192 (19)	−0.0018 (19)
C2	0.067 (3)	0.043 (3)	0.051 (3)	0.004 (2)	0.019 (2)	−0.001 (2)
C3	0.071 (3)	0.057 (3)	0.047 (3)	−0.016 (2)	0.026 (2)	−0.012 (2)
C4	0.089 (4)	0.074 (4)	0.058 (3)	−0.025 (3)	0.022 (3)	−0.006 (2)
C5	0.097 (5)	0.116 (6)	0.076 (4)	−0.052 (4)	0.035 (3)	−0.005 (4)
C6	0.071 (4)	0.139 (6)	0.092 (4)	−0.034 (4)	0.036 (3)	−0.014 (4)
C7	0.063 (3)	0.103 (5)	0.098 (4)	−0.013 (3)	0.039 (3)	−0.011 (3)
C8	0.057 (3)	0.065 (3)	0.061 (3)	−0.012 (2)	0.029 (2)	−0.010 (2)
C9	0.052 (3)	0.057 (3)	0.062 (3)	0.006 (2)	0.021 (2)	−0.008 (2)
C10	0.053 (2)	0.039 (2)	0.046 (2)	−0.001 (2)	0.0214 (19)	−0.0018 (19)
C11	0.063 (3)	0.043 (3)	0.072 (3)	0.011 (2)	0.025 (2)	0.004 (2)
C12	0.089 (3)	0.043 (3)	0.066 (3)	0.006 (2)	0.031 (3)	0.002 (2)
C13	0.099 (4)	0.064 (3)	0.068 (3)	−0.013 (3)	0.035 (3)	0.009 (3)
C14	0.085 (3)	0.090 (4)	0.059 (3)	−0.019 (3)	0.031 (3)	0.013 (3)
C15	0.063 (3)	0.114 (5)	0.079 (4)	−0.009 (3)	0.042 (3)	−0.011 (3)
C16	0.056 (3)	0.105 (4)	0.097 (4)	0.007 (3)	0.038 (3)	−0.009 (3)
C17	0.053 (3)	0.081 (4)	0.090 (4)	0.024 (2)	0.026 (3)	0.013 (3)
C18	0.067 (3)	0.063 (3)	0.077 (3)	0.025 (2)	0.031 (2)	0.015 (2)
C19	0.053 (3)	0.049 (3)	0.069 (3)	−0.010 (2)	0.035 (2)	−0.006 (2)
C20	0.041 (3)	0.085 (4)	0.071 (3)	0.003 (2)	0.005 (2)	0.010 (3)

### Geometric parameters (Å, °)

**Table:**

Co1—N2	1.934 (4)	C4—C5	1.360 (6)
Co1—N2^i^	1.934 (4)	C4—H4	0.9300
Co1—N1^i^	1.956 (4)	C5—C6	1.367 (7)
Co1—N1	1.956 (4)	C5—H5	0.9300
Na1—O6	2.278 (3)	C6—C7	1.363 (7)
Na1—O2	2.329 (3)	C6—H6	0.9300
Na1—O5	2.339 (3)	C7—C8	1.412 (5)
Na1—O4	2.343 (3)	C7—H7	0.9300
Na1—O3	2.348 (3)	C8—C9	1.404 (5)
Na1—O1	2.361 (3)	C9—C10	1.366 (5)
N1—C19	1.162 (5)	C9—H9	0.9300
N2—C20	1.147 (5)	C11—C12	1.488 (5)
O1—C1	1.371 (4)	C11—H11A	0.9700
O1—C18	1.438 (4)	C11—H11B	0.9700
O2—C10	1.369 (4)	C12—H12A	0.9700
O2—C11	1.432 (4)	C12—H12B	0.9700
O3—C12	1.425 (4)	C13—C14	1.494 (6)
O3—C13	1.427 (5)	C13—H13A	0.9700
O4—C14	1.425 (5)	C13—H13B	0.9700
O4—C15	1.427 (5)	C14—H14A	0.9700
O5—C17	1.420 (5)	C14—H14B	0.9700
O5—C16	1.432 (5)	C15—C16	1.493 (6)
O6—H1	0.8393	C15—H15A	0.9700
O6—H3	0.8435	C15—H15B	0.9700
S1—C19	1.610 (5)	C16—H16A	0.9700
S2—C20	1.622 (5)	C16—H16B	0.9700
C1—C2	1.351 (5)	C17—C18	1.496 (5)
C1—C10	1.423 (5)	C17—H17A	0.9700
C2—C3	1.420 (5)	C17—H17B	0.9700
C2—H2	0.9300	C18—H18A	0.9700
C3—C8	1.407 (6)	C18—H18B	0.9700
C3—C4	1.416 (6)		
			
N2—Co1—N2^i^	111.7 (3)	C8—C7—H7	119.7
N2—Co1—N1^i^	108.47 (16)	C9—C8—C3	119.3 (4)
N2^i^—Co1—N1^i^	108.11 (15)	C9—C8—C7	122.0 (5)
N2—Co1—N1	108.11 (15)	C3—C8—C7	118.7 (5)
N2^i^—Co1—N1	108.47 (16)	C10—C9—C8	120.9 (4)
N1^i^—Co1—N1	112.0 (2)	C10—C9—H9	119.5
O6—Na1—O2	103.94 (11)	C8—C9—H9	119.5
O6—Na1—O5	105.07 (12)	C9—C10—O2	126.0 (4)
O2—Na1—O5	133.49 (12)	C9—C10—C1	119.7 (4)
O6—Na1—O4	106.64 (12)	O2—C10—C1	114.2 (3)
O2—Na1—O4	130.18 (12)	O2—C11—C12	108.4 (3)
O5—Na1—O4	73.18 (12)	O2—C11—H11A	110.0
O6—Na1—O3	86.87 (11)	C12—C11—H11A	110.0
O2—Na1—O3	70.98 (10)	O2—C11—H11B	110.0
O5—Na1—O3	145.58 (12)	C12—C11—H11B	110.0
O4—Na1—O3	72.47 (11)	H11A—C11—H11B	108.4
O6—Na1—O1	115.14 (13)	O3—C12—C11	108.7 (3)
O2—Na1—O1	65.28 (9)	O3—C12—H12A	110.0
O5—Na1—O1	69.71 (11)	C11—C12—H12A	110.0
O4—Na1—O1	129.50 (12)	O3—C12—H12B	110.0
O3—Na1—O1	134.41 (11)	C11—C12—H12B	110.0
C19—N1—Co1	178.3 (4)	H12A—C12—H12B	108.3
C20—N2—Co1	172.3 (4)	O3—C13—C14	107.4 (4)
C1—O1—C18	118.8 (3)	O3—C13—H13A	110.2
C1—O1—Na1	115.9 (2)	C14—C13—H13A	110.2
C18—O1—Na1	114.2 (2)	O3—C13—H13B	110.2
C10—O2—C11	118.7 (3)	C14—C13—H13B	110.2
C10—O2—Na1	116.3 (2)	H13A—C13—H13B	108.5
C11—O2—Na1	112.7 (2)	O4—C14—C13	107.4 (3)
C12—O3—C13	113.5 (3)	O4—C14—H14A	110.2
C12—O3—Na1	113.8 (2)	C13—C14—H14A	110.2
C13—O3—Na1	111.6 (2)	O4—C14—H14B	110.2
C14—O4—C15	115.0 (3)	C13—C14—H14B	110.2
C14—O4—Na1	104.7 (2)	H14A—C14—H14B	108.5
C15—O4—Na1	103.3 (3)	O4—C15—C16	107.7 (4)
C17—O5—C16	112.7 (3)	O4—C15—H15A	110.2
C17—O5—Na1	114.6 (2)	C16—C15—H15A	110.2
C16—O5—Na1	111.1 (3)	O4—C15—H15B	110.2
Na1—O6—H1	122.3	C16—C15—H15B	110.2
Na1—O6—H3	128.2	H15A—C15—H15B	108.5
H1—O6—H3	107.3	O5—C16—C15	108.5 (4)
C2—C1—O1	126.3 (4)	O5—C16—H16A	110.0
C2—C1—C10	120.3 (4)	C15—C16—H16A	110.0
O1—C1—C10	113.4 (4)	O5—C16—H16B	110.0
C1—C2—C3	120.7 (4)	C15—C16—H16B	110.0
C1—C2—H2	119.7	H16A—C16—H16B	108.4
C3—C2—H2	119.7	O5—C17—C18	108.5 (3)
C8—C3—C4	118.7 (4)	O5—C17—H17A	110.0
C8—C3—C2	119.0 (4)	C18—C17—H17A	110.0
C4—C3—C2	122.3 (4)	O5—C17—H17B	110.0
C5—C4—C3	120.2 (5)	C18—C17—H17B	110.0
C5—C4—H4	119.9	H17A—C17—H17B	108.4
C3—C4—H4	119.9	O1—C18—C17	106.4 (3)
C4—C5—C6	121.3 (6)	O1—C18—H18A	110.4
C4—C5—H5	119.3	C17—C18—H18A	110.4
C6—C5—H5	119.3	O1—C18—H18B	110.4
C7—C6—C5	120.5 (5)	C17—C18—H18B	110.4
C7—C6—H6	119.8	H18A—C18—H18B	108.6
C5—C6—H6	119.8	N1—C19—S1	178.9 (4)
C6—C7—C8	120.6 (5)	N2—C20—S2	179.2 (5)
C6—C7—H7	119.7		

Symmetry codes: (i) −*x*+1, *y*, −*z*+3/2.

### Hydrogen-bond geometry (Å, °)

**Table:**

*D*—H···*A*	*D*—H	H···*A*	*D*···*A*	*D*—H···*A*
O6—H1···S2^ii^	0.84	2.62	3.458 (3)	173
O6—H3···S1^iii^	0.84	2.57	3.389 (3)	165

Symmetry codes: (ii) *x*, −*y*+1, *z*−1/2; (iii) −*x*+3/2, −*y*+1/2, −*z*+1.
